# Severe familial dilated cardiomyopathy in a young adult due to a rare LMNA mutation: a case report

**DOI:** 10.1093/ehjcr/ytae423

**Published:** 2024-08-14

**Authors:** Adam M Belcher, Frank H Annie, Sarah Rinehart, Ahmad Elashery, Muhammad Amer

**Affiliations:** CAMC Institute for Academic Medicine, Charleston Area Medical Center, 3044 Chesterfield Ave, Charleston, WV 25304, USA; CAMC Institute for Academic Medicine, Charleston Area Medical Center, 3044 Chesterfield Ave, Charleston, WV 25304, USA; CAMC Department of Cardiology, Charleston Area Medical Center, 3200 MacCorkle Ave SE, Charleston, WV 25304, USA; CAMC Department of Cardiology, Charleston Area Medical Center, 3200 MacCorkle Ave SE, Charleston, WV 25304, USA; CAMC Department of Cardiology, Charleston Area Medical Center, 3200 MacCorkle Ave SE, Charleston, WV 25304, USA

**Keywords:** Dilated cardiomyopathy, LMNA, Genetic disease, Heart failure, Case report

## Abstract

**Background:**

Familial dilated cardiomyopathy prognosis and disease progression vary greatly depending upon the type of genetic mutation. Family history and genetic testing are paramount in developing the best treatment plan for a patient. However, with rare or novel mutations, the significance may be unknown. Regarding this, the following case report highlights the importance of vigilance and suspicion when treating a patient with a variant of unknown significance. Additionally, it shows the importance of thoroughly investigating the family history of cardiovascular disease.

**Case summary:**

A 25-year-old Caucasian male was found to have a right bundle branch block and dilated cardiomyopathy upon presentation to the emergency department. Later testing showed that the dilated cardiomyopathy was due to an incredibly rare lamin A/C (LMNA) gene mutation, R349L. Despite treatment with a maximum-tolerable medication regimen and an automatic implantable cardioverter-defibrillator, the patient continued to decline and required a heart transplant.

**Discussion:**

This case provides more information on the severity of this specific *LMNA* mutation that has only been documented once before. Of note, the time from the initial emergency department visit to the heart transplant was approximately 2 years. Given the patient’s young age and rapid disease progression, in addition to a strong family history of sudden cardiac death, the significance of this mutation should not be understated. The additional knowledge gained from this case report can be used to aid in timely interventions and prognosis evaluation.

**Learning points**

1. Investigating family history and published literature plays an important role in determining variant pathogenicity and prognosis of familial dilated cardiomyopathy.
2. Several factors, including New York Heart Association class, conduction abnormalities, sudden cardiac death risk, disease progression, and current guidelines, should be considered when determining optimal treatment strategies.
3. This case presented a few key challenges: young onset of the disease, a genetic variant with only one previously published case report and scant *in vitro* work, and quick progression of the disease despite using optimal therapies. Close follow-up and timely interventions were paramount in this case.

## Background

Non-ischaemic dilated cardiomyopathy (DCM) is estimated to occur in approximately 1:250, and of those, approximately 30–50% are assumed to be familial.^1^ Lamin A/C (LMNA) gene mutations are a known cause of familial DCM, conveying a high risk of sudden cardiac death (SCD) at an estimated rate as high as 46%.^2^ In a retrospective study of 49 families of patients with DCM in Colorado, USA, and Italy, carriers of an *LMNA* mutation were more likely to be younger (22–32 years old) than non-carriers at the time of diagnosis. They were also much more likely to have conduction abnormalities, a heart transplant, and SCD.^3^ The European Society of Cardiology recommends consideration for a cardioverter-defibrillator in patients with DCM and a confirmed disease-causing *LMNA* mutation coupled with other clinical risk factors.^4,5^

## Summary figure

**Figure:**
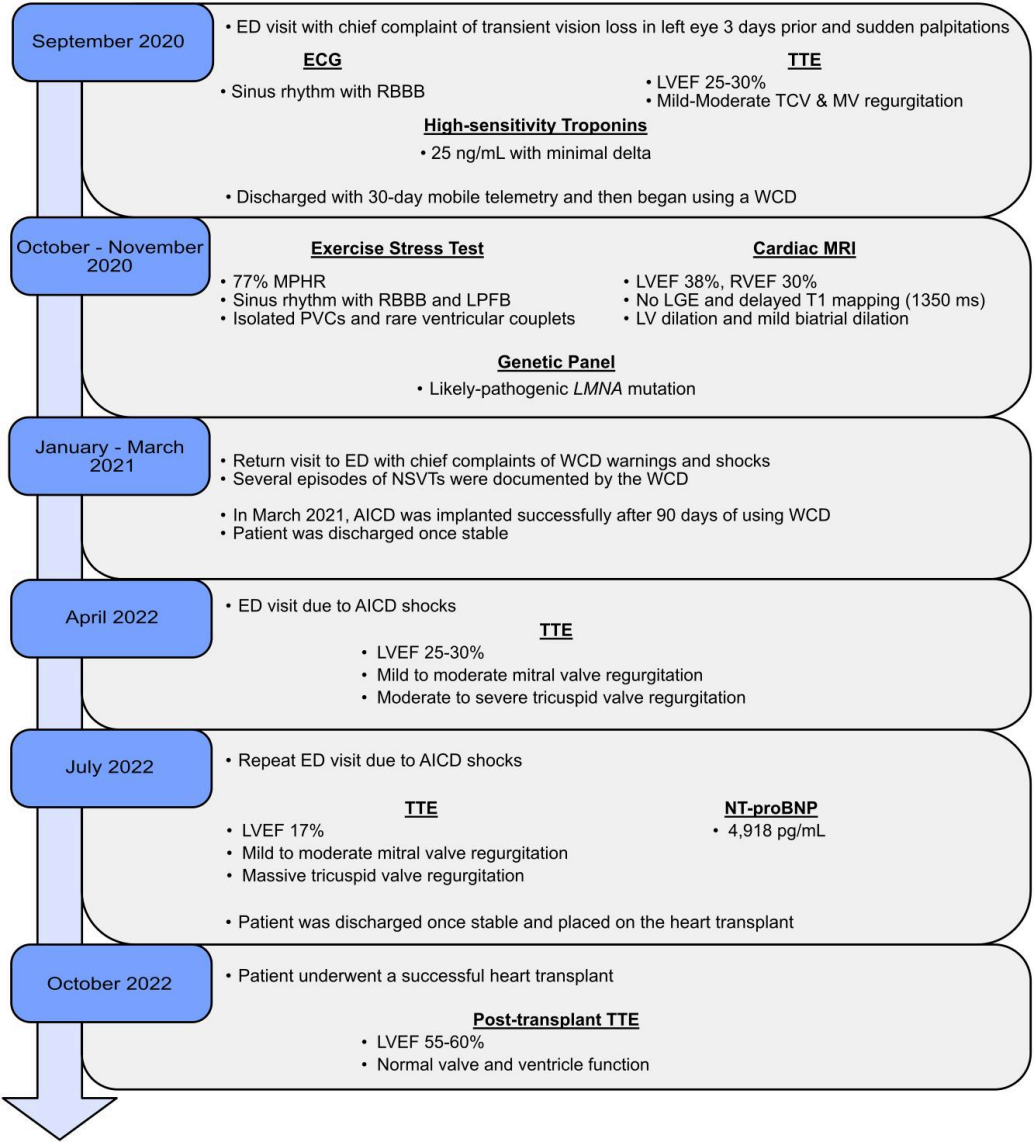


## Case report

A 25-year-old Caucasian landscaper presented to the emergency department (ED) with the chief complaints of lifelong recurrent, sudden onset palpitations, and an episode of vision loss that occurred 3 days prior and lasted for 30 min before resolving on its own. He reports developing a headache after his vision returned. Physical examination was unremarkable, with a temperature of 36.4°C, blood pressure of 112/72 mmHg, and peripheral pulse rate of 70 b.p.m.

The patient had no significant past medical history or known cardiovascular risk factors aside from previously stated episodes of palpitations. Initial family history revealed that the patient’s mother died in her 30s from DCM. Further investigation elucidated a family history very significant for heart failure and SCD.

At presentation, ophthalmic migraine, reduced blood flow, and stroke were considerations for the sudden vision loss of the left eye. Panic disorder is not a likely cause of his palpitations, given his long history of these palpitations without any known history of psychological issues or triggers. Given the patient’s extensive family history of early-onset heart failure and SCD, young age, and palpitations, genetic cardiomyopathy was the primary consideration.

**Figure 1:**
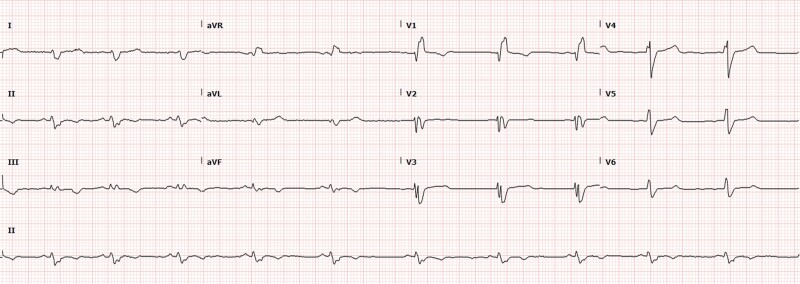
Intake electrocardiogram. Baseline 12-lead electrocardiogram with normal sinus rhythm and PR interval with a right bundle branch block and repolarization changes.

**Figure 2:**
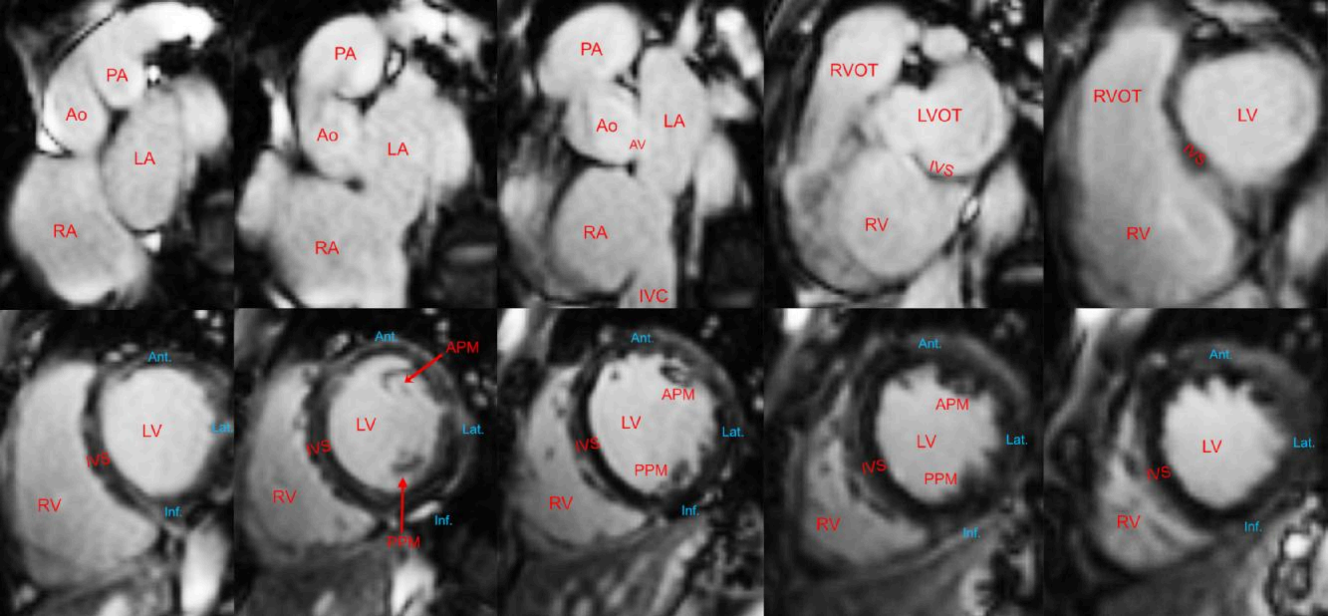
Cardiac magnetic resonance imaging. Cardiac magnetic resonance imaging showing cross-sectional short-axis images with no late gadolinium enhancement. Ao, aorta; PA, pulmonary artery; LA, left atrium; RA, right atrium; IVC, inferior vena cava; AV, aortic valve; RVOT: right ventricle outflow tract; LVOT, left ventricle outflow tract; IVS, interventricular septum; LV, left ventricle; RV, right ventricle; Ant., anterior left ventricle wall; Lat., lateral left ventricle wall; Inf., inferior left ventricle wall; APM, anterolateral papillary muscle; PPM, posteromedial papillary muscle.

**Figure 3:**
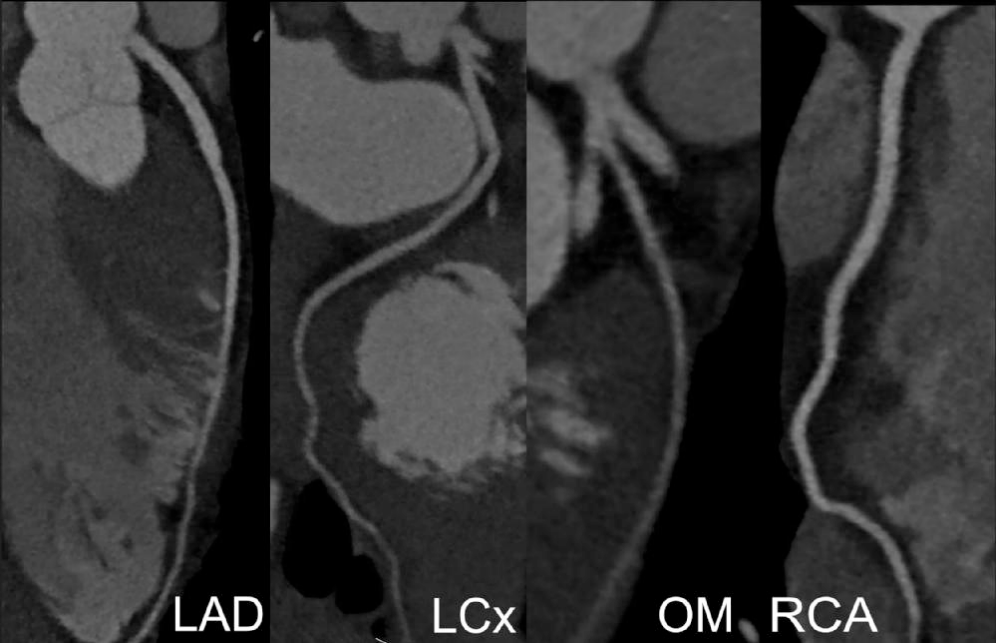
Coronary computed tomography angiography. The coronary computed tomography angiography showed no obvious signs of arterial disease or other abnormalities. LAD, left anterior descending; LCx, left circumflex; OM, obtuse marginal; RCA, right coronary artery.

Initial testing at presentation included a 12-lead electrocardiogram (ECG) and a high-sensitivity troponin test. The ECG showed sinus rhythm with a normal PR interval and a right bundle branch block (RBBB) (*Figure 1*). High-sensitivity troponin was 25 pg/mL (normal ≤ 20 pg/mL) with minimal delta. Head computed tomography (CT) and magnetic resonance imaging (MRI) were unremarkable. Transthoracic echocardiogram findings were significant for mildly dilated, severely diffuse hypokinetic left ventricle with an ejection fraction (EF) of 20–25%, a grade III diastolic dysfunction with elevated atrial pressure, and mild–moderate mitral and tricuspid valve regurgitation (Supplementary material online, *Video S1*). Right ventricle cavity size was mildly increased, and systolic function was mildly reduced. A cardiac MRI showed a left ventricular EF (LVEF) of 38% and a right ventricular EF of 30%, no evidence of prior infarction or late gadolinium enhancement, and an increased T1 mapping signal of 1350 ms (*Figure 2*) (Supplementary material online, *Video S2*). Coronary CT angiography revealed no coronary artery disease or anomaly (*Figure 3*). A genetic testing panel for cardiomyopathy was performed per the guidelines concerning newly diagnosed cardiomyopathy. At this time, he was classified as a class II heart failure following the New York Heart Association (NYHA) guidelines.

He was then discharged from the hospital with a maximum-tolerable guideline-directed medical therapy for heart failure (aspirin 81 mg, atorvastatin 40 mg, losartan 25 mg, and metoprolol 25 mg) and a wearable cardioverter-defibrillator (WCD). He was instructed to promptly return to the ED if anything changed. He was also scheduled for an exercise stress test and a follow-up appointment with his cardiologist.

The exercise stress test was non-diagnostic due to his heart rate only reaching 77% of the maximum predicted heart rate, and the test was stopped after 12 min due to fatigue. Blood pressure increased from 96/67 to 130/62 mmHg, and heart rate increased from 70 to 150 b.p.m. during the stress test. Isolated premature ventricular contractions and rare ventricular couplets were seen during the test.

**Figure 4:**
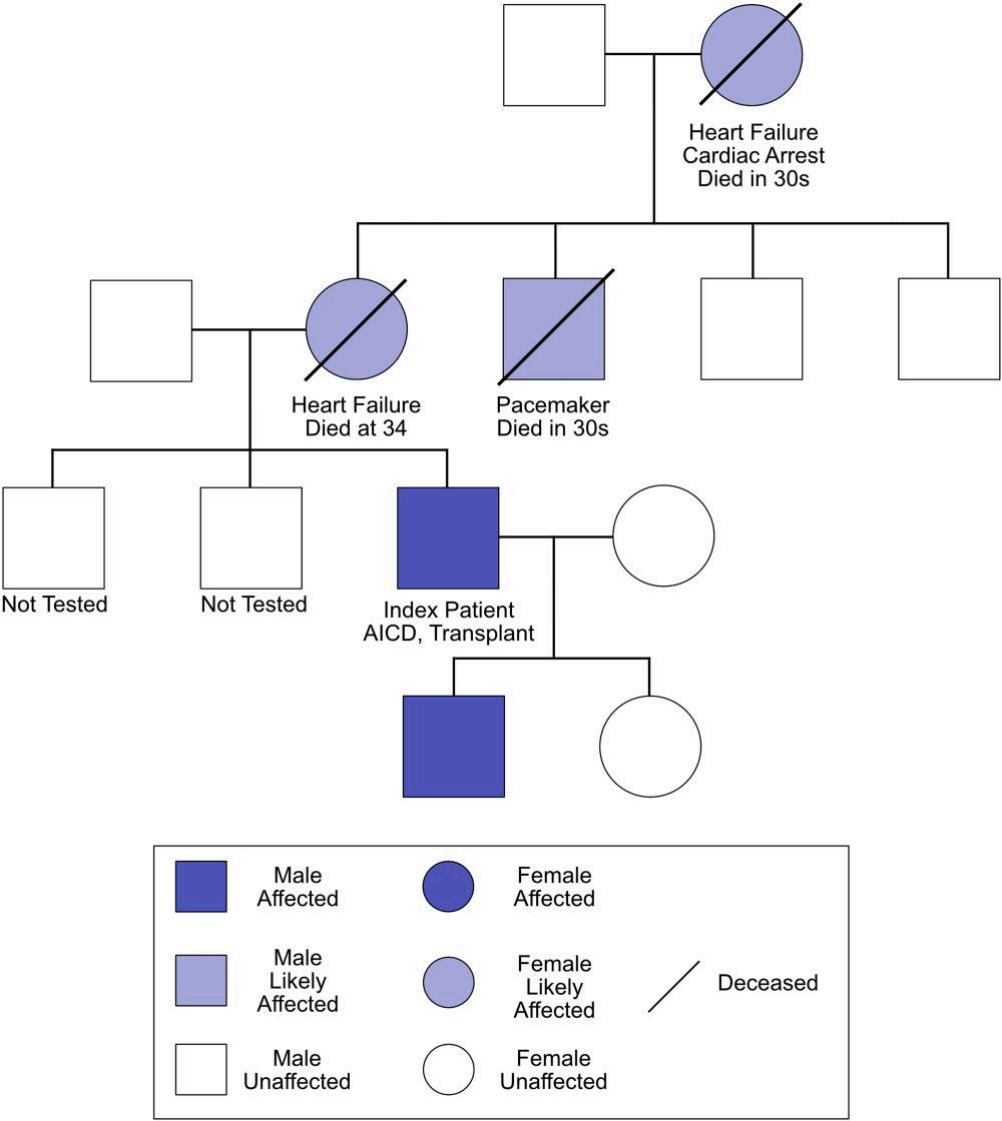
Genetic cascade screening. *LMNA* mutation pedigree chart of biological family detailing R349L carrier, or presumed carrier, status, and applicable cardiovascular health.

Genetic testing confirmed that the patient was heterozygous for a likely pathogenic missense single nucleotide mutation (rs58789393) in exon 6 codon 349 (R349L) of the *LMNA* gene, which confirms our suspicion of familial DCM. Further in-depth screening of the patient’s family history revealed a potential association between the mutation and heart failure (*Figure 4*).

**Figure 5:**
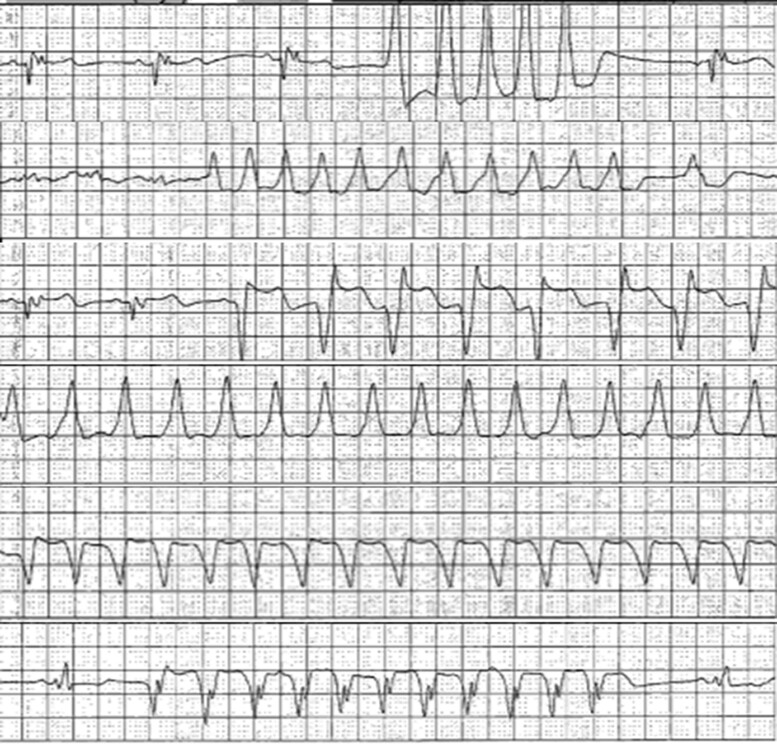
Wearable cardioverter-defibrillator notification. Wide complex, non-sustained ventricular tachycardia with the wearable cardioverter-defibrillator.

A few weeks after the initial ED visit, the patient presented to the ED due to episodes of ventricular tachycardia detected by the WCD, followed by WCD discharge. The patient stated that the WCD detected ventricular tachycardia during physical activity (*Figure 5*). This led him to reduce his workload and physical activity. Upon follow-up, it was determined that an automatic implantable cardioverter-defibrillator (AICD) was the best course of action over cardiac resynchronization therapy given his continual decline despite 3 months of optimal therapy, non-ischaemic cardiomyopathy with non-sustained ventricular tachycardia, NYHA class II, RBBB with sinus rhythm, and a strong family history of SCD. Additionally, he was instructed to stop taking his losartan for 2 days and then begin taking sacubitril/valsartan (24/26 mg) twice a day.

In the following year, the patient’s health declined. He reported increased shortness of breath, especially when climbing stairs, and he had to leave his occupation as a landscaper. Given this continued decline, he was referred to an out-of-state facility for further evaluation. At that time, he was found to have a massive amount of tricuspid regurgitation, mild–moderate mitral valve regurgitation, an LVEF of 17%, worsened function in both ventricles, and a significantly elevated N-terminal B-type natriuretic peptide level at 4918 pg/mL (normal 0–449 pg/mL) (Supplementary material online, *Video S1*). He was placed on a transplant list and underwent a successful transplant a few months later. The new heart showed a 55–60% LVEF with normal valve and ventricle function. He has since remained stable and is closely followed by cardiology.

## Discussion

The R349L mutation has, to the best of our knowledge, only been documented once. In 2004, Hermida-Prieto *et al*.^6^ described a case of severe familial cardiomyopathy in a mother (index patient), and her identical twin daughters referred to their cardiomyopathy clinic in Spain that were heterozygous for the R349L mutation. The index patient was reportedly symptomatic at age 31 and received a transplant at age 36, and the twin daughters were symptomatic at age 17 and received transplants at age 18 and 20. The R349L mutation is upstream of the nuclear localization signal which is associated with a more severe phenotype.^6^ No family history of musculoskeletal disorders or progeroid syndromes was reported. Interestingly, musculoskeletal disorders and progeroid syndromes have been seen in a different mutation of the same codon (R349W).^7^

This mutation has been classified as ‘likely pathogenic’ based on the previously reported case^8^ and *in silico* pathogenicity models.^9^ The R349L variant has not yet been detected in large-scale sequencing projects or aggregate databases. Therefore, allele frequency within a population or ethnicity is unknown.

In younger patients with a strong family history of heart failure and SCD, genetic testing aids in the accurate diagnosis of familial DCM. The more severe phenotypes, such as the one seen in the R349L mutation, indicate a need for prompt treatment. This is evident in this case, where the patient continued to decline despite an aggressive treatment strategy utilizing an AICD and maximum-tolerable medications. Considering the patient’s continual decline, a heart transplant was ultimately needed. The rapid progression of disease in this patient’s family, as well as the one other documented family, shows the poor prognosis and severity associated with this mutation.

The presented case highlights the importance of genetic screening in patients with a strong family history of SCD and newly diagnosed cardiomyopathy. This case also highlighted some challenges faced in managing cardiomyopathy, such as identifying cardiomyopathy type, the progression of symptoms despite medical interventions, and the radical changes required in the patient’s physical activity and lifestyle. Ultimately, the patient underwent a successful heart transplant, and he has continued to improve and regain his previous quality of life.

## Supplementary Material

ytae423_Supplementary_Data

## Data Availability

All data is presented within the article and in the associated online Supplementary material.

## Lead author biography

Adam Belcher is a research scientist at the Charleston Area Medical Center Institute for Academic Medicine. His primary interests are cardiovascular disease and molecular biology research.

## Notes

**Consent:** In accordance with the standards established by the Committee on Publication Ethics (COPE) and the ICMJE recommendations, we have obtained patient consent to publish this manuscript and its associated contents, and we have taken steps to protect the patient’s identity.

**Ethics approval:** None declared.
