# Supplementary material for: Severe familial dilated cardiomyopathy in a young adult due to a rare LMNA mutation: a case report
Source: Eur Heart J Case Rep. 2024 Aug 14;8(9):ytae423. doi: 10.1093/ehjcr/ytae423 (PMC11375576; doi:10.1093/ehjcr/ytae423)
Supplement: ytae423_Supplementary_Data [file ytae423_supplementary_data.zip › Supplemental Video File Legends.docx]

**Supplemental Video File Legends**

**Video S1:** A cardiac MRI taken in November 2020 assessed morphological function in three different views: four chamber, left ventricular outflow tract, and right ventricular outflow tract.

**Video S2:** An initial transthoracic echocardiogram and a follow-up in April 2022 showing flow dynamics in the parasternal long-axis view.
